# Contamination inside CT gantry in the SARS-CoV-2 era

**DOI:** 10.1186/s41747-020-00182-1

**Published:** 2020-10-01

**Authors:** João Matos, Francesco Paparo, Marco Mori, Alessio Veneziano, Marina Sartini, Maria Luisa Cristina, Gian Andrea Rollandi

**Affiliations:** 1 DISSAL–Department of Health Sciences, University of Genoa, Via Antonio Pastore, 1, 16132 Genova, Italy; 2 S.C. Radiodiagnostica, EO Ospedali Galliera, Mura delle Cappuccine, 14, 16128 Genova, Italy; 3 S.C. Laboratorio Analisi, EO Ospedali Galliera, Mura delle Cappuccine, 14, 16128 Genova, Italy; 4 Independent Researcher, Plymouth, UK; 5 S.S.D. U.O. a Direzione Universitaria Igiene Ospedaliera, EO Ospedali Galliera, Mura delle Cappuccine, 14, 16128 Genova, Italy

**Keywords:** COVID-19, Patient safety, Safety management, Severe acute respiratory syndrome coronavirus 2, Tomography scanners (x-ray computed)

## Abstract

We investigated whether the internal gantry components of our computed tomography (CT) scanner contain severe acute respiratory syndrome 2 (SARS-CoV-2) ribonucleic acid (RNA), bacterial or fungal agents**.** From 1 to 27 March 2020, we performed 180 examinations of patients with confirmed SARS-CoV-2 infection using a dedicated CT scanner. On 27 March 2020, this CT gantry was opened and sampled in each of the following components: (a) gantry case; (b) inward airflow filter; (c) gantry motor; (d) x-ray tube; (e) outflow fan; (f) fan grid; (g) detectors; and (h) x-ray tube filter. To detect SARS-CoV-2 RNA, samples were analysed using reverse transcriptase-polymerase chain reaction (RT-PCR). To detect bacterial or fungal agents, samples have been collected using “replicate organism detection and counting” contact plates of 24 cm^2^, containing tryptic soy agar, and subsequently cultured. RT-PCR detected SARS-CoV-2 RNA in the inward airflow filter sample. RT-PCR of remaining gantry samples did not reveal the presence of SARS-CoV-2 RNA. Neither bacterial nor fungal agents grew in the agar-based growth medium after the incubation period. Our data showed that SARS-Cov-2 RNA can be found inside the CT gantry only in the inward airflow filter. All remaining CT gantry components were devoid of SARS-CoV-2 RNA.

## Key points

- Severe acute respiratory coronavirus 2 (SARS-CoV-2) ribonucleic acid (RNA) was found inside the computed tomograohy (CT) gantry.
- SARS-CoV-2 RNA was found solely in the inward airflow filter sample.
- This filter could be a barrier to SARS-CoV-2 dissemination.
- Neither bacteria nor fungi were cultured from CT gantry samplings.

## Background

Many computed tomography (CT) scanners are equipped with potent air-cooling systems to accomplish x-ray tube heat control. The drawbacks associated with air cooling include the potential contamination of both CT gantry and examination room that comes with the required substantial airflow (up to 4,000 m^3^ air/h) [1]. During the severe acute respiratory syndrome coronavirus 2 (SARS-CoV-2) pandemic, CT scans have been used extensively [2], as we also did at our institution [3]. It is conceivable that contamination of the CT gantry or suite may occur due to the high volume of these examinations. We took this occasion to investigate the presence of SARS-CoV-2 ribonucleic acid (RNA), bacterial, and fungal agents in various components inside the CT gantry.

## Methods

We analysed a 16-slice CT scanner (CT lightspeed-16™, GE Healthcare, Milwaukee, WI, USA). From 1 to 27 March 2020 (when sampling was conducted), this CT scanner performed 180 consecutive examinations of patients with confirmed SARS-CoV-2 infection. No patients undergoing invasive ventilation were scanned. After each study, we used surface disinfection with 62–71% ethanol or 0.1% sodium hypochlorite. Passive air exchange was performed for 30–60 min, as well as a professional cleansing of the dedicated CT suite after each 8-h shift. During the aforementioned period, the CT gantry was not opened or suctioned, and none of the sampled internal components were disinfected.

**Fig. 1:**
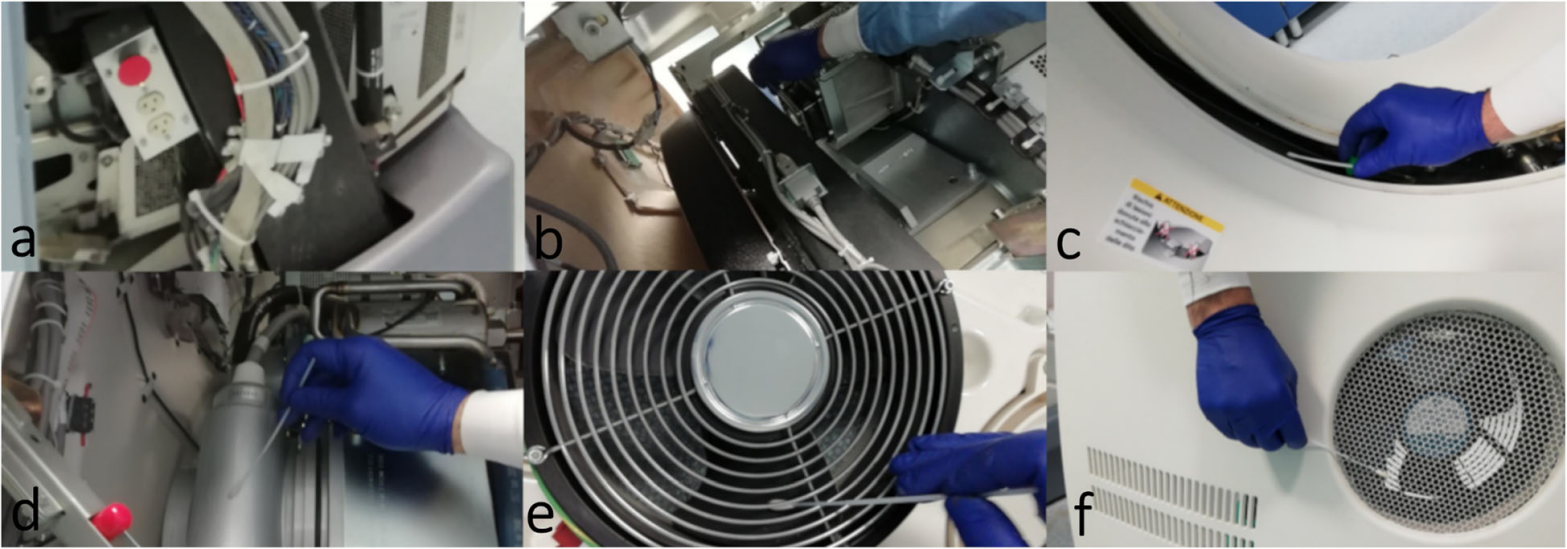
Computed tomography gantry internal component sampling. **a** Gantry case. **b** Inward airflow filter. **c** Gantry motor. **d** x-ray tube. **e** Outflow fan. **f** Fan grid

To test the internal microbiologic contamination, the CT gantry was opened and sampled in each of the following zones: (a) gantry case; (b) inward airflow filter; (c) gantry motor; (d) x-ray tube; (e) outflow fan; (f) fan grid; (g) detectors; and (h) x-ray tube filter. Guided by dust deposition sites, swabs have been rotated slightly in such a way as to utilise every part available for sampling and have been swept in close parallel lines. Each sample covered a total area of 100 cm^2^. The sampling procedure is illustrated in Fig. 1. Samplings were performed at 10:00 am. In that morning, the CT suite underwent a deep professional cleansing at 7:00 am. Afterward, four patients with confirmed SARS-CoV-2 infections underwent CT, with surface disinfection as described above between each of the examinations.

To detect SARS-CoV-2 RNA, each sample was inserted into a collection vial containing 1–3 mL of viral transport media and subsequently underwent reverse transcriptase-polymerase chain reaction (RT-PCR) assay (Simplexa™ COVID-19, DiaSorin Molecular, Cypress, CA, USA). Besides, samples from each site have been collected using “replicate organism detection and counting” contact plates of 24 cm^2^, containing tryptic soy agar. Aliquots have been cultured, and analysis has been carried out to measure total microbial counts at 22 °C and 37 °C, total mycotic count, and to identify specific microorganisms.

## Results

**Fig. 2:**
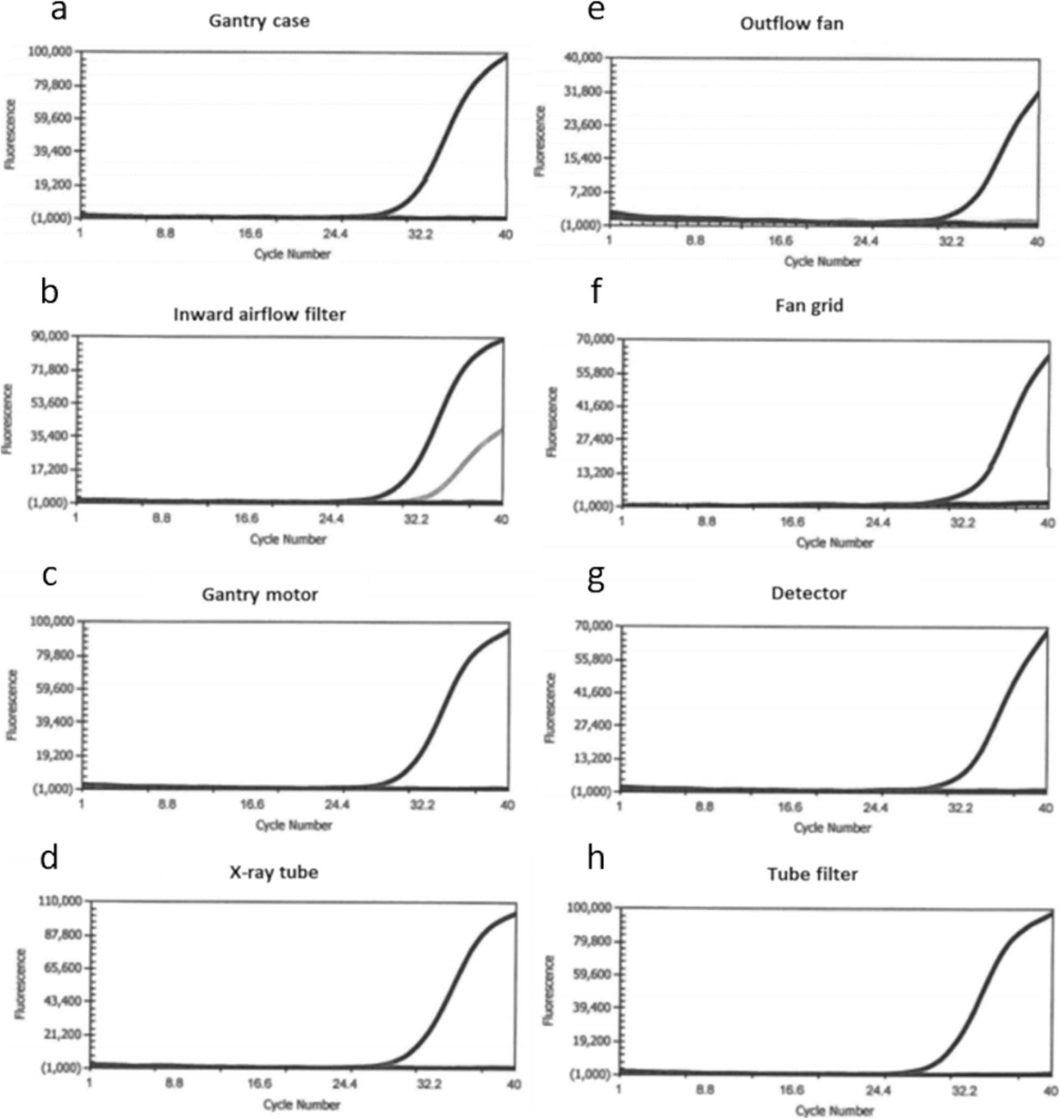
Fluorescence *versus* cycle plot shows amplification of samples (**a**–**h**). After reverse-transcription of ribonucleic acid (RNA), deoxyribonucleic acid (DNA) in each sample was also amplified with every cycle. In the example above, only sample from **b** contained RNA. Thus, it is the only plot that shows an increase in fluorescence (DNA amplification)

RT-PCR detected SARS-CoV-2 RNA in the zone (b) sample, *i.e*., the inward airflow filter. RT-PCR of remaining gantry zone samples did not reveal the presence of SARS-CoV-2 RNA. The fluorescence curves for each sampling zone is shown in Fig. 2. No bacterial or fungal agents grew in the agar-based growth medium after the incubation period.

## Discussion

In this brief study, we sampled different zones in a CT scanner that was dedicated and heavily used during the SARS-CoV-2 outbreak at our institution.

There has been much concern regarding the survival of SARS-CoV-2 on inanimate surfaces. Earlier SARS-CoV research concluded that coronaviruses might persist in inanimate surfaces for up to 9 days. It was also found that commonly used disinfectants are effective in inactivating the virus [4]. Notably, the internal part of the CT gantry cannot routinely be disinfected and may pose a risk to personnel performing CT maintenance. Likewise, drawing vast amounts of air from the lower portion of the gantry CT system, near the floor, and expelling it out of the upper part of the gantry chamber, may cause dispersion of viral particles inside the CT gantry and in the CT suite room.

Our study found SARS-CoV-2 RNA only in the inward airflow filter. All other gantry sampling sites were free of SARS-CoV-2 RNA. These results are encouraging since this filter may act as a partial barrier to the virus. No viral RNA was detected in the dust of internal case and particularly on the outflow fan system (propeller and grid), meaning the absence of contamination in both the internal components of the CT gantry and the CT suite room. Besides, no bacterial or fungal agents were cultured. Even after 26 days of intensive use, conventional sanitisation measures were most probably successful in preventing large-scale contamination of the CT scanner.

The study had some limitations. First, the analysis was performed at only one time point. Second, we did not perform exhaustive sampling of the CT gantry; though, the sampled areas were the ones most likely to have microbial agents since it was where dust deposited. Finally, RT-PCR has shown suboptimal sensitivities in previous studies, and therefore some areas containing SARS-CoV-2 RNA could have been missed.

In conclusion, SARS-Cov-2 RNA was found internally in the CT gantry only in the inward airflow filter. All remaining CT gantry zones were devoid of SARS-CoV-2 RNA. No bacterial or fungal cultures were obtained from internal CT gantry sampling zones.

## Data Availability

Data will be made available on reasonable request.

## Abbreviations

CT: Computed tomography
RNA: Ribonucleic acid
RT-PCR: Reverse transcriptase-polymerase chain reaction
SARS-CoV-2: Severe acute respiratory syndrome coronavirus 2
